# Pre-operative carotid artery screening in patients undergoing coronary artery bypass grafting

**DOI:** 10.1186/1749-8090-10-S1-A20

**Published:** 2015-12-16

**Authors:** Ana-Catarina Pinho-Gomes, Rana Sayeed

**Affiliations:** 1 Department of Cardiac Surgery, John Radcliffe Hospital, Oxford, OX3 9AA, UK

## Background/Introduction

Stroke remains the major non-cardiac complication of coronary artery bypass surgery (CABG). Severe carotid artery disease is associated with a fourfold increase in the risk of post-operative stroke and this is the rationale for offering pre-operative carotid artery screening to these patients.

## Aims/Objectives

We aim to assess the compliance with the ESC/EACTS guidelines for Myocardial Revascularisation (2014) regarding pre-operative carotid artery disease screening and estimate the clinical impact of adhering only to class-I evidence-based recommendations.

## Method

The medical records of all the patients who underwent CABG in our unit between 1st November and 31st December 2014 were retrospectively reviewed.

## Results

A total of 506 patients underwent CABG during the study period and 492 were included for analysis. 203 carotid artery Doppler ultrasound scans were performed. CAD screening was performed in 63/115 of the patients who met with class-I recommendations (history of stroke/transient ischaemic attack or carotid bruit) and 184/440 of the patients who met with class-IIa recommendations (age over 70 years and/or peripheral artery disease and/or multi-vessel coronary artery disease).

There were 2 post-operative strokes, both in patients without CAD detected on pre-operative screening. Asynchronous carotid artery revascularisation was performed in 5 patients (4 prior to and 1 following CABG).

Restricting carotid artery screening to class-I evidence-based recommendations criteria would have decrease the number of Doppler ultrasound scans from 203 to 115, without missing patients with CAD who actually required revascularisation.

## Conclusion

Adherence to class-I evidence-based recommendations for carotid artery screening would generate major efficiency savings and streamline pre-operative assessment of patients undergoing CABG.

